# The Hospital World

**Published:** 1911-05-20

**Authors:** 


### THE HOSPITAL WORLD.

### Congratulations for a Carnival Secretary.

The annual success of the carnival in aid of the Ilford Emergency Hospital, and the part which it has played in raising the necessary funds, was mentioned in our issue of May 21 last year. To Mr. E. R. Fyson, its energetic Hon. Secretary, the popularity of the hospital carnival has been largely due, and the occasion of his silver wedding has been taken advantage of to express the congratulations and thanks of the hospital's supporters. Mr. Fyson, as editor of the *Ilford Recorder*, has been able to assist the hospital not only at carnival time.

### A Royal Portrait for the Gravesend Hospital.

At the Gravesend Hospital on Wednesday last, in the presence of a large gathering of governors, Mrs. Raoul H. Foa, of Hollywell Park, unveiled a portrait of H.R.H. Princess Alexander of Teck in one of the women's wards and, with the gracious permission of Her Royal Highness, formally named the ward "Princess Alice" after her. The portrait bears a plate commemorating the visit made by the Princess to the hospital in July last year, which was the means of raising over a thousand pounds. It will be a perpetual and graceful reminder of Her Royal Highness' work for the Gravesend Hospital, which is happy to be one of the few institutions to possess a portrait of perhaps the most enthusiastic hospital worker among the Royal Family. There is no need to emphasise here how much their Royal Highnesses of Teck have done for hospitals since the late Prince Francis and the present Prince Alexander have become known as hospital officials.

### An Event at the West Ham Hospital.

At the West Ham and Eastern General Hospital on Wednesday last week a very interesting little ceremony took place. Sir John Reynolds Roberts, a very old and liberal supporter of the hospital, presented to Miss Ough, the ex-matron of the hospital, a purse containing a cheque for over fifty guineas, subscribed for by the staff, the committee, and a few of her personal friends, as a memento of her fifteen years' work at West Ham Hospital, during the last ten years of which she acted as matron. The chair was taken by Mr. Thos. Alex. Cook, the Chairman of the Hospital, who spoke of the great tact and excellent qualities as matron which Miss Ough had exercised throughout the whole of this period. Sir John Reynolds spoke of the great work the hospital was doing, and said that he had watched Miss Ough's career with the very greatest interest, and had pleasure in testifying to the splendid manner in which she had carried out her duties and the great improvements that had taken place in the hospital under her matronship. He also complimented the committee on having got together the enormous amount of money for the extension to the building which has become so necessary. Dr. P. J. S. Nicoll, Senior Honorary Physician to the Hospital, explained that the Patroness, Her Grace the Duchess of Marlborough, would have been present if possible, but had sent to him that morning a very beautiful silver and tortoiseshell jewel case suitably inscribed, which she had asked him to present on her behalf to Miss Ough, wishing her prosperity, health, and happiness in her new career. Tea was served in the grounds, and as many of the staff as could be spared from their duties mixed with the visitors, and a most enjoyable reunion was carried through.

